# Trait analysis in a population of the Greater Butterfly-orchid observed through a 16-year period

**DOI:** 10.3389/fpls.2023.1213250

**Published:** 2023-08-08

**Authors:** Richard M. Bateman, Katherine M. Stott, David F. Pearce

**Affiliations:** ^1^ Jodrell Laboratory, Royal Botanic Gardens Kew, Surrey, United Kingdom; ^2^ Independent Researcher, Brighton, Sussex, United Kingdom

**Keywords:** climate change, cross-correlation, developmental constraint, Greater Butterfly-orchid, life history, natural selection, *Platanthera chlorantha*, time-series

## Abstract

A large English population of the temperate tuberous Greater Butterfly-orchid, *Platanthera chlorantha*, was monitored through a 16-year period. Each June the number of flowering plants was counted and 60 flowering plants were measured *in situ* for four morphological traits, selected for both ease of measurement and their contrasting contributions to the life history of the species. Trait data were tested annually in pairwise combinations for individual plants, before mean values throughout the study period were regressed and cross-correlated against each other and against local data for four meteorological parameters. Labellar spur length proved to be more constrained than either flower number or stem height, and rarely yielded statistically significant correlations with other traits, whereas the three remaining traits reliably showed modest but significant correlations. Mean values and coefficients of variation differed only modestly among years and showed few of any meaningful trends. Spring rainfall and insolation had no detectable effect on traits of plants flowering that June; instead, they impacted on trait expression during the following year, presumably as a result of differential resourcing of replacement tubers formed during the previous year. High spring rainfall in year t–1 increased leaf area and stem height in year t, whereas the widely fluctuating number of flowering plants was highest in years immediately following those characterised by relatively dry and/or sunny springs. The “decision” to flower is taken during the previous summer, though it may be modified through winter/spring abortion of above-ground organs. The proportion of the population electing to flower is the only measured parameter that impacts significantly on annual reproductive output, emphasising the under-rated difficulty of evolving through directional selection. Any attempt to predict the behaviour of plant species in response to climate change must integrate information on demography with that on life history, habitat preference and intimate symbioses.

## Introduction

Medium- to long-term scientific experiments remain all too uncommon in a scientific realm that has become increasingly dependent on, and so constrained by, short-term funding. In a recent meta-analysis of 822 diverse animal populations, White (2019) concluded that on average a continuous demographic monitoring period of 15.9 years was needed for meaningful conservation assessment of populations (an issue also addressed by Field et al., 2007; Magurran et al., 2010; Hughes et al., 2017). Those relatively few long-term experiments that have survived the test of time have reliably delivered data of exceptional value (Frost et al., 2006; Silvertown et al., 2006; Bateman, 2011). Many such examples involve the establishment of sets of “permanent” plots, aiming to monitor long-term ecological change in natural habitats, both terrestrial (e.g. Plotkin et al., 2000; Condit et al., 2005) and marine (e.g. Worm et al., 2008). Others conduct annual sampling to explore the effects of contrasting agricultural treatments (Moss et al., 2004; Silvertown et al., 2006) or the life-history demographics of particular species (e.g. Wells, 1991; Wells et al., 1998; Hutchings, 2010). In yet other cases, populations of chosen species have been monitored to capture fluctuations in traits of particular interest, though most commonly the data represent only reproductive effort and reproductive success rather than encompassing a broader suite of morphological traits (cf. Grant and Grant, 2002; Tremblay et al., 2006; Grant and Grant, 2008; Olaya-Arenas et al., 2011; Laughlin, 2023). They also tend to be medium-term rather than long-term, representing a period of time closer to a decade than a century.

Using methods first established in the UK by Terry Wells (Wells, 1967, et seq.), European temperate orchids have long provided some of the best case-studies of demographic studies of plant species involving data-gathering through at least a decade (Wells, 1967; Willems, 1978; Wells and Cox, 1989; Wells, 1991; Willems and Bik, 1991; Hutchings et al., 1998; Waite and Farrell, 1998; Wells et al., 1998; Carey et al., 2002; Willems, 2002; Hutchings, 2010; Jacquemyn et al., 2010; Bódis et al., 2019). One such study, concerning the demographics of a single population of the tuberous terrestrial orchid *Anacamptis morio*, has operated continuously since 1978 (Wells et al., 1998; Stroh, 2019). However, most relevant investigations followed through many years the annual behaviours of particular plants within the targeted populations (plus, in some cases, percentage fruit set) at the expense of monitoring multiple morphological traits. Other projects that have instead focused on several selected traits have tended to limit data collection to only a single flowering season or at best to very few seasons (e.g. Sletvold et al., 2012; Miano et al., 2015; Chapurlat et al., 2020).

**Figure 1:**
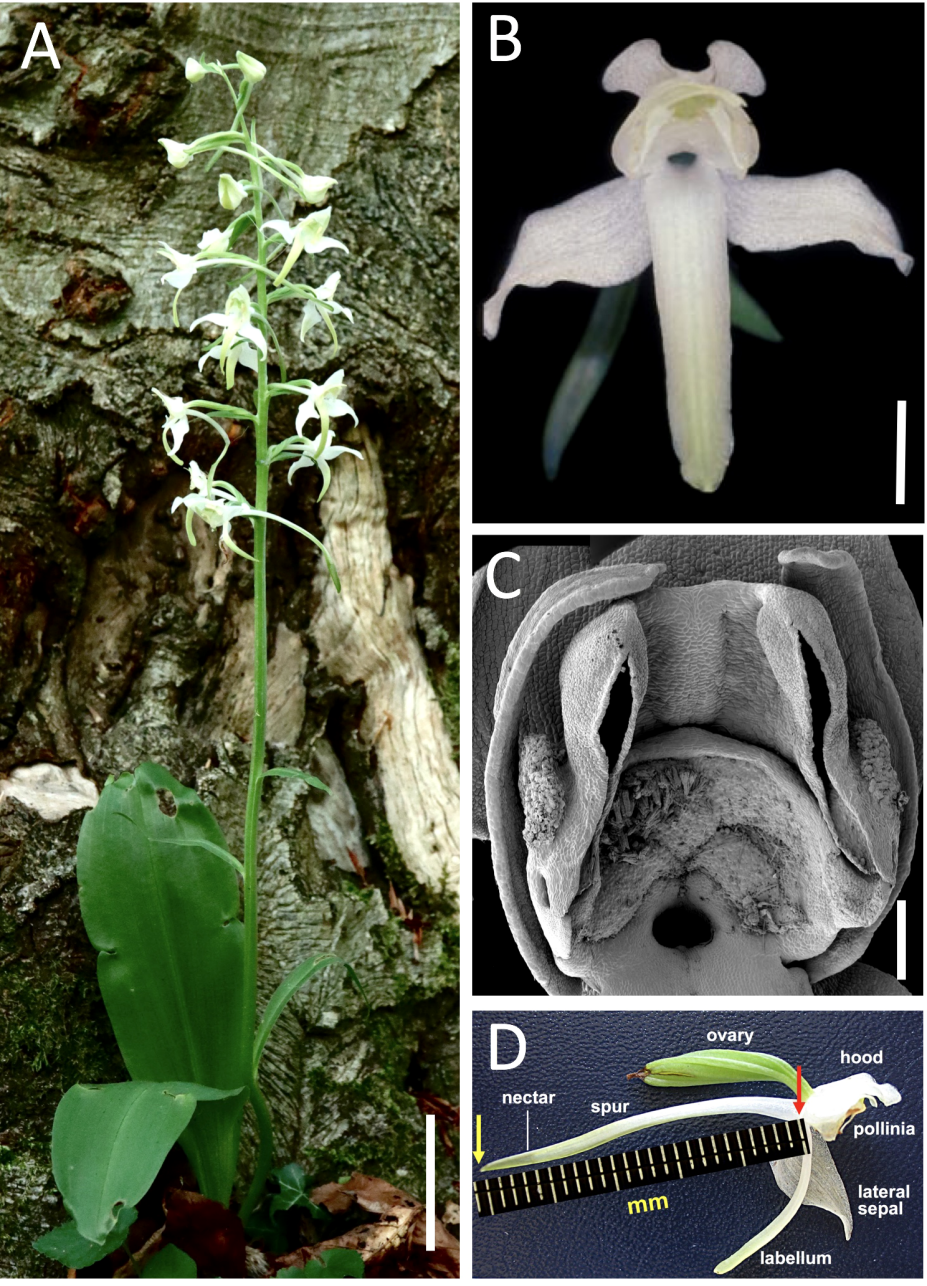
**(A–D)** Morphology of the study species – the Greater Butterfly-orchid, *Platanthera chlorantha*. **(A)** Typical plant in full flower, featuring the multi-flowered inflorescence and paired elliptical basal leaves. **(B)** Uniformly white, moth-pollinated flower. **(C)** Scanning electron micrograph showing details of the gynostemium and spur entrance. **(D)** Lateral view of flower highlighting the long, highly nectariferous labellar spur. Images: **(A, B)**, Richard Bateman; **(C)**, Paula Rudall; **(D)**, Roy Sexton and Richard Bateman. Scales: **(A)** = 50 mm; **(B)** = 5 mm, **(C)** = 1 mm.

Here, we report variation in four disparate morphological traits, together with annual number of flowering plants, in a large well-established population of the tuberous terrestrial orchid *Platanthera chlorantha* (Custer) Rchb. (Greater Butterfly-orchid: **Figure 1**) located in southern England. In contrast with most previous studies, traits in our study population were sampled annually through a period of 16 successive years, albeit through random sampling of flowering plants rather than through tracking of particular marked plants through successive years. The resulting data are tested through both standard and cross-correlations, both among each other and with long-term data on rainfall, sunshine, temperature and frost days obtained from a nearby meteorological station. The four morphological traits measured – labellar spur length, number of flowers per inflorescence, stem height, total leaf area – were selected because they differ in scale, play contrasting roles in the life history of the plant, and are likely to operate under contrasting degrees of developmental and/or selectional constraint (e.g. Bateman et al., 2013; Bateman and Rudall, 2019). The interplay between the chosen characters therefore interested us from an evolutionary viewpoint. However, it is the shorter-term response of the population to variation in local climate that has obvious implications for better understanding biotic responses to broader-scale, human-induced climate change (e.g. Foster et al., 2014; Fournier-Level et al., 2016; Keating-Bitonti and Chang, 2018; White, 2019; Bateman, 2022; Kirschner et al., 2022).

## Materials and methods

### Study population

Our study population of *Platanthera chlorantha* occupies a strip of calcareous downland and gradually encroaching scrub, situated at the foot of the north-facing slope of Wolstonbury Hill. Managed by the National Trust, the Hill forms a salient along the South Downs chalk escarpment of East Sussex, 10 km North of Brighton, England (N 50°54’, W 0°10’; *ca* 103 m asl). The Wolstonbury population was first utilised scientifically in 2007, when it contributed to a citizen science project designed to explore variation across Europe in spur length in both *P. chlorantha* and *P. bifolia* (Bateman and Sexton, 2008; Bateman and Sexton, 2009; Bateman et al., 2012). In 2008, the initial short-term project was expanded to include a further three morphological variables plus annual counts of the number of flowering plants (**Table 1**).

**Table 1:** Values of the four variables measured for the present study during the period 2007–2022, together with corresponding data for local climatic variables.

Year	Rain Mar-June*	Sun Mar-June	Temp Mar-June	Frost Nov-April	No. fl. plants†	Sample size	Spur length (mm)		Flower no.		Stem height (cm)		Leaf area (cm^2^)	
	mm	hours	°C	days			Mean	CV	Mean	CV	Mean	CV	Mean	CV†
**2022**	122	798	15.6	2	295	60	30.4	8.8	14.6	26.5	39.4	15.6	51.6 (0)	38.5
**2021**	142	805	13.9	17	348	60	28.8	9.5	12.0	27.4	32.1	15.3	34.6 (0)	47.9
**2020**	161	1009	16.0	4	308	60	26.6	10.4	14.7	21.5	34.4	17.1	44.5 (0)	41.3
**2019**	201	805	15.4	8	141	60	29.4	7.6	12.6	26.9	33.7†	30.2	39.2 (0)	41.9
**2018**	245	831	15.2	22	167	60	30.1	10.0	13.2	27.3	35.3	13.7	50.3 (0)	45.8
**2017**	197	765	16.4	13	72	60	29.2	5.6	12.1	29.6	35.6	16.7	48.4 (0)	44.9
**2016**	327	707	14.9	8	162	60	29.4*	7.9	12.0	39.7	35.7	22.6	44.1 (7)*	63.0
**2015**	177	822	15.0	14	73	60	29.9	9.6	14.1	29.2	39.4	19.3	66.4 (0)*	63.5
**2014**	213	836	15.7	0	117	60	30.4	9.6	14.3	37.8	41.0	25.1	56.8 (0)*	60.2
**2013**	258	627	12.8	14	172	60	29.7	10.2	14.7	27.9	41.6	16.1	69.8 (3)	40.6
**2012**	437	810	14.9	14	315	60	28.0*	12.9	13.7	29.6	36.6	17.8	52.2 (2)	49.5
**2011**	176	929	15.9	24	340	60	27.6*	10.0	13.5	31.0	33.8	17.4	45.0 (0)	58.4
**2010**	175	937	14.7	27	350	60	28.6*	9.3	14.3*	38.7	33.4	16.4	48.4 (3)*	63.6
**2009**	202	917	15.7	21	45	45	29.4*	9.2	14.2	33.2	42.0	19.5	69.8 (2)*	44.7
**2008**	307	799	15.6	9	56	25	28.9	9.6	13.3	36.6	37.7	23.2	55.2 (20)	60.2
**2007**	280	839	16.2	4	36	36	27.5	11.8	13.2	28.3	–	–	–	–
**2006**	273	823	14.0	13	–	–	–	–	–	–	–	–	–	–
**2005**	168	797	14.6	13	–	–	–	–	–	–	–	–	–	–
**Series Mean**	229	827	15.1	12.6	187	NA	29.0	9.5	13.5	30.7	36.8	19.1	51.8	50.9

Parenthetic numbers for mean leaf area show the percentage of plants that possessed only one leaf (two plants bore three leaves in 2015). Asterisks indicate the presence of at least one outlying data point for that year (traits) or for the entire time series (climatic variables); the dagger is the equivalent notation for strong bimodality within the data. Dashes indicate the absence of data, 'NA' denotes 'Not Applicable'. The insolation figure for 2022 is provisional.

By the end of data collection in 2022, the Wolstonbury population of *P. chlorantha* was estimated by us to consist of at least 500 mature individuals. However, the population extends over an area exceeding one hectare; this fact, combined with habitat disturbance resulting from constant public access, soon dissuaded us from pursuing more than superficial attempts to monitor the long-term performance of individual plants. We were therefore obliged to focus on annual fluctuations in the overall performance of the population, an approach that at least offered the advantage of reliably delivering a statistically robust sample size (e.g. Lindley, 1997).

### Morphometric data collection

Our study was entirely non-destructive. Morphometric data were collected *in situ* in mid-June of 16 successive years between 2007 and 2022. The chosen date of measurement varied from June 1st in unusually early seasons to June 23rd in unusually late seasons, the mean date being June 11th. Following an initial count of numbers of flowering plants, only plants approximating full flower were included in the otherwise randomly selected annual sample of 60 plants (though only 36, 25 and 45 plants were measured in 2007, 2008 and 2009, respectively – a period when encroaching brambles were gradually cleared from the site by conservation volunteers). In total, 886 data-sets were acquired, collectively yielding 3472 morphological data points.

The four traits selected by us reflect a desire for relative ease of measurement combined with a desire to examine as many properties of the plants as possible using as few traits as possible. Many prior morphometric studies performed by RB and colleagues on populations of a wide range of European orchid species, typically scoring approximately 50 characters per plant (e.g. Bateman and Rudall, 2011; Bateman et al., 2013), had already identified consistent patterns of variation among contrasting traits in several European orchid genera, including *Platanthera* (Bateman et al., 2012; Bateman et al., 2013).

The strongly nocturnally scented white flowers of *P. chlorantha* are typical of orchids pollinated predominantly by moths (**Figures 1A–C**). The precise length of the extensive nectar-rich spur is widely regarded as influencing the spectrum and efficiency of pollinating insects (e.g. Claessens et al., 2008; Claessens and Kleynen, 2011; Esposito et al., 2017). It is therefore important that it should be measured with precision (**Figure 1D**); details of the optimal procedure were given by Bateman and Sexton (2008). Because orchid flowers reliably diminish in size from the base to the apex of the inflorescence (Bateman and Rudall, 2006), for each plant the spur was measured on a flower located midway along the inflorescence. The number of flowers (including any unopened buds) on the inflorescence was recorded, being the trait that largely determines the reproductive output of the individual. From 2008 onward, we also measured stem height from ground level to the tip of the inflorescence (**Figure 1A**). This character not only determines inflorescence density but also the height at which the flowers are presented to prospective pollinators and, subsequently, the distance over which the resulting dust-seeds are likely to disperse from the dehisced seed-pods.

The final trait to be measured was the maximum width of each expanded leaf (as seen in the lower part of **Figure 1A**, almost all mature plants produce a pair of expanded basal leaves, though occasionally a comparatively weak plant will produce only one). Measuring this trait was simpler than attempting to estimate in the field the aggregate surface area of the leaves, which would have constituted the best proxy for photosynthetic potential. In order to gain a more precise yardstick for leaf area, we therefore measured the maximum width and length of 22 leaves sampled in 2020, before using pixel numbers in scanned outlines to determine their precise areal extent. We were thus able to determine that multiplying maximum width by length yielded on average a figure that exceeded the true areal extent of the leaf by 30%, and that the squared value of the maximum width yielded a figure that was 60.9 ± 8.5% of the true areal extent. This figure formed the basis of our *post hoc* conversion of measured actual width to yield an estimate of the areal extent of each leaf, subsequently summating the values of the (usually) paired leaves to reflect the likely relative photosynthetic potential of the plant in question.

### Meteorological data acquisition

The monthly figures summarized in **Table 1** for rainfall, sunshine and maximum daytime temperature during the main growth period of March–June throughout the 16-year period of study were downloaded from internet repositories for the viable meteorological stations located closest to Wolstonbury. Data for rainfall were obtained from Plumpton Agricultural College, located 6 km east of Wolstonbury Hill and representing a broadly similar aspect and altitude. Unfortunately, the nearest site providing reliable sunshine and temperature data, along with total frost days per winter, was the meteorological station at Eastbourne, 36 km ESE of Wolstonbury, where the lower altitude and coastal location are likely to have yielded sunshine figures somewhat higher, and frost figures marginally lower, than the number of hours experienced by the Wolstonbury plants. Comparison of March–June rainfall data from the two stations gathered since 2005 showed rainfall to be on average 29% lower at Eastbourne, though intriguingly, the respective annual means converged significantly during the final four years of the present study.

### Data analysis

Microsoft Excel was used to calculate mean, sample standard deviation and coefficient of variation for each of the nine variables included in **Table 1**, for each of the 16 years, together with mean values for the entire time-series. Linear correlations were performed in Excel (v15.40 and v16.70) before being double-checked and presented in Red Rock’s Deltagraph (v7.1.3) package. All six possible pairwise combinations of the four morphological traits were plotted for all plants measured in each year of study.

This exercise was then repeated for annual mean values for each trait, together with the number of plants that flowered annually in the population, thus requiring ten pairwise combinations. The comparisons were conducted not only on a same-year basis (year t) but also with the data staggered to allow comparisons with the previous year (t–1) and the year before that (t–2), thereby increasing the number of valid comparisons to 65.

Although this project was conceived partly to simply explore the properties of the four contrasting morphological traits, it also aimed to address more ambitious questions, such as whether climatic conditions and/or photosynthetic potential during the critical period of one annual growth cycle may impact on plant performance during the following annual growth cycle. Therefore, annual mean values for the four morphological traits across the 16 years of study, together with number of flowering plants within the population, were plotted against aggregate March–June rainfall (at Plumpton) and aggregate March–June sunshine, mean maximum monthly temperature, and number of frost days (at Eastbourne).

Tabulated *r* values for all correlations were subsequently subjected to two-tailed *p* tests (with 58 degrees of freedom for samples of 60 plants in the case of within-year comparisons, and 14 degrees of freedom for among-year comparisons of the 16 successive years of mean values).

Seeking a more sophisticated check on conclusions drawn from these simple linear regressions, we then repeated our time-displaced comparison of each trait with each of the four categories of meteorological data through autocorrelation – specifically, applying temporal cross-correlation methods to covariance values in order to generate sliding dot products of values scaled to a range between –1 and +1 relative to the average for the recording period (e.g. Derrick and Thomas, 2004; Tahmasebi et al., 2012).

## Results

**Table 1** presents mean values and coefficients of variation for the four morphological traits in each year of the study, together with the total number of flowering plants in the population and the yearly figures for March–June aggregate rainfall, insolation, mean maximum daily temperature, and number of frost days. The accumulated data are perhaps most remarkable for their consistency through time and relatively low levels of variance across all four morphological traits. There is evidently a clear stepwise increase in typical within-year coefficients of variation among the four traits, from spur length (*ca* 10%) through stem height (*ca* 20%) and flower number (*ca* 30%) to leaf area (*ca* 50%). However, the coefficient for leaf area was inevitably exaggerated through being presented as an area rather than a linear or meristic measure (coefficients of variation for the original linear measure of leaf width approximated 20%). Similar contrasts in levels of variance were evident in the climatic variables; frost days and spring rainfall were more variable than insolation, which was in turn more variable than temperature.

Within yearly figures, outlying individual data points proved surprisingly uncommon (affected data-sets are asterisked in **Table 1**). Occasional plants produced unusually short spurs in five of the 16 years, and in a further five years occasional plants had unusually large leaves, while in 2010 one plant bore a remarkable total of 36 flowers – three times the long-term average. The population appeared to perform more consistently from 2017 onward, after which coefficients of variation exceeding 35% for flower number and 60% for leaf area no longer occurred, nor were any further plants recorded as bearing only one leaf (**Table 1**). Significant bimodality was identified in only one distribution of individual values – that for stem height as measured in 2019, when a dearth of stems in the range 23–30 cm resulted in an atypically high coefficient of variation. Among the extrinsic climatic variables summarized in the four left-hand columns of **Table 1**, spring rainfall shows an unusually large – arguably outlying – value for 2012, and 2013 was notable for a relatively cold and cloudy spring. Monitored through the 16-year study period, the number of flowering plants – essentially a response variable reflecting interactions in the recent past between a plant with its environment – fluctuated greatly, yielding a coefficient of variation of 64%. This property also showed bimodality; flowering frequency was much higher in two phases each of three consecutive years (2010–2012 and 2020–2022).

**Figure 2:**
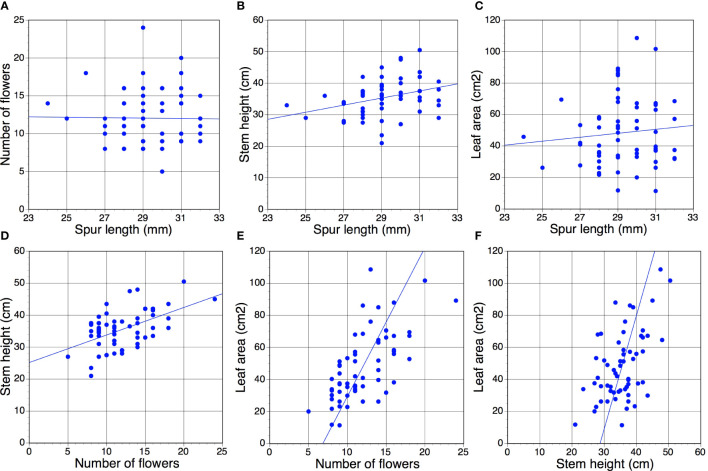
**(A–F)** All six possible year t versus year t pairwise correlations of the four morphological traits measured for the present study, based on a sample of 60 individuals scored for the exemplar year 2017 (**Table 1**). Statistical significance of *r* values: **(D–F)**, *p <*0.01; **(B)**, *p <*0.05 (barely); **(A, C)**, *p* >>0.05 (**Table 2**).

**Table 2** presents *r* values for the six possible pairwise comparisons of the four morphological traits; data for an exemplar year (2017) are also represented in graphical form in **Figure 2**. Of the 91 pairwise combinations reported, 51 (56%) yielded *p* values below 0.05, 45 (49%) of which also yielded *p* values below 0.01. Comparisons involving spur length – the trait of lowest variance – rarely yielded statistically significant results. Only in three years toward the end of the study (2017 onwards) did *p <*0.01 relationships emerge from spur data; all three positive correlations reflected comparisons of spur length versus stem height. In contrast, almost all of the trait comparisons that did not involve spur data yielded positive correlations of *p <*0.01 that, moreover, were similar in magnitude when averaged across 15 years. The only such results failing the significance test were the flower number versus stem height comparisons for 2011 and 2020 – the year of highest spring rainfall, when stem height correlated especially poorly with the other morphological traits (**Tables 1**, **2**).

**Table 2:** Values of, and bivariate regressions between, the four variables measured for annual samples of individual plants during the period 2007–2022.

Year	Sampled Plants	Spur v. Flower	Spur v. Stem	Spur v. Leaf	Flower v Stem	Flower v. Leaf	Stem v. Leaf
	*n*	*r*	*r*	*r*	*r*	*r*	*r*
**2022**	60	-0.055	0.187	-0.089	***0.440***	***0.444***	***0.435***
**2021**	60	0.148	***0.383***	0.228	***0.418***	***0.482***	***0.540***
**2020**	60	-0.063	0.212	0.105	0.219	***0.613***	**0.292**
**2019**	60	0.232	***0.383***	**0.261**	***0.543***	***0.431***	***0.442***
**2018**	60	-0.118	***0.404***	0.141	***0.555***	***0.496***	***0.488***
**2017**	60	-0.045	**0.324**	0.100	***0.516***	***0.656***	***0.517***
**2016**	60	-0.089	0.126	0.170	***0.770***	***0.656***	***0.713***
**2015**	60	0.130	0.190	-0.031	***0.578***	***0.391***	***0.439***
**2014**	60	0.110	**0.330**	0.152	***0.642***	***0.760***	***0.735***
**2013**	60	-0.055	**0.310**	**0.268**	***0.421***	***0.481***	***0.567***
**2012**	60	-0.243	0.161	0.032	***0.479***	***0.706***	***0.480***
**2011**	60	-0.226	0.164	0.176	0.184	***0.351***	***0.553***
**2010**	60	-0.243	0.130	0.032	***0.539***	***0.760***	***0.692***
**2009**	45	0.045	0.270	0.134	***0.647***	***0.634***	***0.623***
**2008**	25	0.253	0.354	0.359	***0.888***	***0.841***	***0.889***
**2007**	36	0.032	–	–	–	–	–
**Series Mean**	NA	0.148	0.239	0.148	0.547	0.615	0.622

Boldface values are *p <*0.05, boldface italicised values are *p <*0.01. Dashes indicate the absence of data.

**Table 3** presents *r* values for pairwise comparisons of annual mean values for the four morphological traits, plus annual number of flowering plants, plotted against each other. This comparison was initially conducted for the same year of observation, t, but then one of the two columns of data was shifted by first one year (t–1) and then two years (t–2) to determine whether one trait could impact on another in future growing seasons. As we were unable to realistically envisage such an influence extending over a period of more than one year, we performed the t–2 comparisons primarily to act as de facto null hypotheses, anticipating that no t–2 comparison would yield a statistically significant result.

**Table 3:** Values of *r* for regressions of annual means of the four morphological traits plus number of plants flowering in the population against each other.

	Spur length	Flower number	Stem height	Leaf area	No. of flowering plants
**Spur length, t**	NA	0.000	**0.547**	0.389	-0.346
**Spur length, t–1**	0.247	-0.055	0.407	0.279	-0.122
**Spur length, t–2**	-0.451	0.000	0.063	-0.155	0.263
**Flower number, t**	0.000	NA	**0.562**	**0.612**	0.148
**Flower number, t–1**	0.100	-0.032	0.155	0.032	0.032
**Flower number, t–2**	**-0.595**	0.219	-0.122	-0.032	0.442
**Stem height, t**	**0.547**	**0.562**	NA	***0.902***	-0.325
**Stem height, t–1**	0.095	-0.032	0.277	0.173	-0.032
**Stem height, t–2**	**-0.675**	0.363	-0.089	0.000	0.412
**Leaf area, t**	0.389	**0.612**	***0.902***	NA	**-0.546**
**Leaf area, t–1**	0.100	0.032	0.345	0.228	-0.148
**Leaf area, t–2**	-0.521	0.310	0.224	0.195	0.321
**No. flowering plants, t**	-0.346	0.148	-0.325	**-0.538**	NA
**No. flowering plants, t–1**	-0.359	0.164	-0.421	-0.292	**0.556**
**No. flowering plants, t–2**	0.032	0.000	-0.405	-0.319	0.114

Boldface values are *p <*0.05, italicised boldface values are *p <*0.01. Year t versus t comparisons are identical vertically and horizontally. 'NA' denotes 'Not Applicable'.

**Figure 3:**
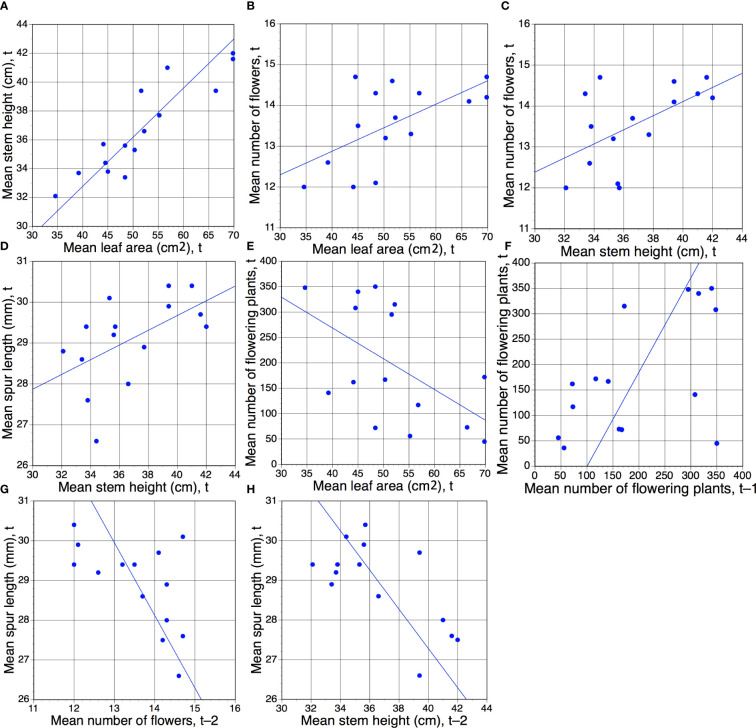
**(A–H)** Eight pairwise combinations of annual mean morphological characters plus annual total number of flowering plants (**Table 1**) that proved to be statistically significant. Of these eight regressions, five represent comparisons for the same year of measurement (t), but one **(F)** involves year t–1 and two **(G, H)** involve year t–2. Statistical significance of *r* values: **(A)**, *p <*0.01; **(B–H)**, *p <*0.05 (**Table 3**).

The resulting table of 75 cells contains 65 unique *r* values (the five year t comparisons involving the same trait are void and a further five year t × year t comparisons are reciprocal duplicates). Only eight of the 65 pairwise comparisons yielded values that were statistically significant; all eight are presented in **Figure 3**. Five are year t × year t comparisons, the first four of which are positive correlations (**Figures 3A–D**) that merely echo the four equivalent positive correlations detected between the same pairs of traits when analysed annually at the level of individual plants (**Figure 2**). Superficially less readily explained are the negative correlation between mean leaf area and the number of plants flowering in the same year (**Figure 3E**), and the fact that a year with many flowering plants is likely to be followed by another year with many flowering plants (**Figure 3F**). Even more obscure are the reasons why years of relatively short mean spur lengths (and higher than normal coefficients of variation) are often followed two years later by relatively tall stems (**Figure 3H**) that bear relatively large numbers of flowers (**Figure 3G**), thereby contradicting our initial assumption that no t versus t–2 comparison would prove statistically significant.

**Table 4** presents *r* values for pairwise comparisons of both spring rainfall and spring sunshine (treated as independent variables) against annual mean values for the four morphological traits plus number of plants flowering within the population. Values for trait comparisons with mean spring maximum daily temperature and number of frost days are also given, but neither achieved statistical significance with any of the recorded traits. Initial analyses compared data for the same year (t), but two further rounds of regression were performed that compared the five dependent variables with each of the meteorological variables for the previous year (t–1) and the year before that (t–2). Our intention was to test prior hypotheses such as whether photosynthetic activity (using leaf surface area as a proxy) in one year might impact on the performance of the population during the following year. As we were unable to realistically envisage such an influence extending over a period of two years, we performed the t–2 comparisons primarily to act as a de facto null hypothesis, anticipating (correctly) that no t–2 comparison would yield a statistically significant result.

**Table 4:** Values of *r* for regressions of annual means of the four morphological traits plus number of plants flowering in the population against four meteorological variables (total annual March–June rainfall, insolation, and mean maximum daily temperature, plus annual number of frost days) treated as independent variables through 16 years (15 years for stem height and leaf area).

	Spur length	Flower number	Stem height	Leaf area	No. of flowering plants
**Rainfall, t**	-0.141	-0.182	0.122	0.148	-0.202
**Rainfall, t–1**	-0.100	0.152	**0.537**	**0.607**	**-0.594**
**Rainfall, t–2**	0.326	0.277	0.232	0.221	-0.176
**Insolation, t**	**-0.543**	0.319	-0.308	-0.195	0.305
**Insolation, t–1**	-0.464	-0.182	**-0.546**	-0.371	**0.615**
**Insolation, t–2**	-0.141	0.251	0.164	0.032	0.437
**Daily temp., t**	-0.259	-0.089	-0.095	-0.184	-0.292
**Daily temp., t–1**	-0.176	-0.219	-0.390	-0.084	0.138
**Daily temp., t–2**	0.032	-0.032	-0.126	-0.286	0.338
**Frost, t**	-0.077	-0.063	-0.152	0.055	0.286
**Frost, t–1**	-0.173	0.100	-0.032	-0.259	0.476
**Frost, t–2**	-0.487	0.330	0.000	0.126	0.214

Boldface values are *p <*0.05, no values are *p <*0.01. Degrees of freedom = number of comparable years minus 2.

**Figure 4:**
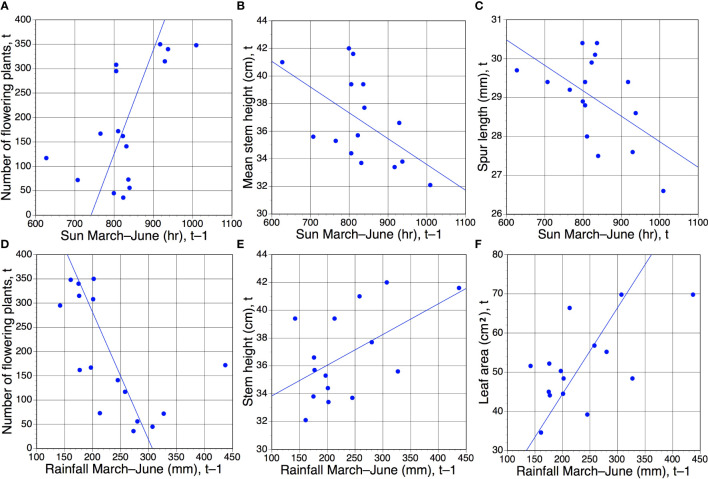
**(A–E)** Those six of the 30 pairwise combinations of morphological characters plus annual number of flowering plants versus March–June rainfall and sunshine (**Tables 1**, **2**) that proved to be statistically significant. Of these six regressions, only one **(C)** compares data from the same year of measurement (t); in the remainder, the meteorological data precede the morphological/phenological data by one year (t–1). Statistical significance of *r* values: **(A–F)**, all *p <*0.05 (**Table 4**).

The six pairwise comparisons that did yield statistically significant results, all approximating similar values of *p* (**Table 4**), are shown graphically in **Figures 4A–F**. Surprisingly, only one same-year (t) comparison yielded a significant result, specifically, unusually high levels of spring sunshine tended to correlate with small but statistically significant (*p <*0.05) reductions in mean spur length by the time of peak flowering in June (**Figure 4C**). In contrast, five regressions based on t–1 comparisons yielded statistically significant results (**Table 4**; **Figures 4A, B, D–F**). When the population was subjected to elevated spring rainfall its component plants tended to produce larger leaves and taller stems during the following year (**Figures 4E, F** **)**. In contrast, a relatively sunny spring tended to result in shorter stems in the subsequent year (**Table 4**; **Figure 4B**). Five of the six statistically significant correlations coincided with dot product cross-correlation values exceeding 0.095 (**Table 5**); the exception was the negative relationship between stem height and insolation in year t–1, which failed the cross-correlation test. In contrast, unlike direct correlation, cross-correlation implied a positive relationship between number of flowering plants and insolation in year t–2 (**Table 5**).

**Table 5:** Values of dot product cross-correlation coefficients of annual means of the four morphological traits plus number of plants flowering in the population against figures for total annual March–June rainfall and insolation, treated as independent variables through 16 years (15 years for stem height and leaf area).

	Spur length	Flower number	Stem height	Leaf area	No. of flowering plants
**Rainfall, t**	-0.0237	-0.0043	0.0207	0.0298	-0.0537
**Rainfall, t–1**	0.0262	0.0379	0.0971	0.1231	-0.1454
**Rainfall, t–2**	0.0401	0.0626	0.0139	0.0218	-0.0562
**Insolation, t**	-0.0994	0.0783	-0.0701	-0.0555	0.0890
**Insolation, t–1**	-0.0766	-0.0360	-0.0077	-0.0786	0.1903
**Insolation, t–2**	-0.0234	-0.0373	0.0306	-0.0122	0.1530
**Leaf area, t**	0.0800	0.1686	0.1710	NA	-0.1884
**Leaf area, t–1**	0.0665	0.0315	0.0383	NA	-0.1066
**Leaf area, t–2**	-0.0276	-0.0130	-0.0254	NA	-0.0760

'NA' indicates 'Not Applicable'.

Perhaps the most striking t–1 trends concerned the number of plants flowering within the population. This variable proved to be somewhat bimodal; for 10 of the 16 years, between 36 and 172 plants flowered, whereas the remaining six years (2010–2012 and 2020–2022) achieved more impressive totals of between 295 and 350 flowering plants (**Table 1**). Those six most floriferous years occurred one year after six of the eight driest springs (**Figure 4D**) and one year after all four of the sunniest springs (**Figure 4A**), in both cases resulting in statistically significant trends.

**Figure 5:**
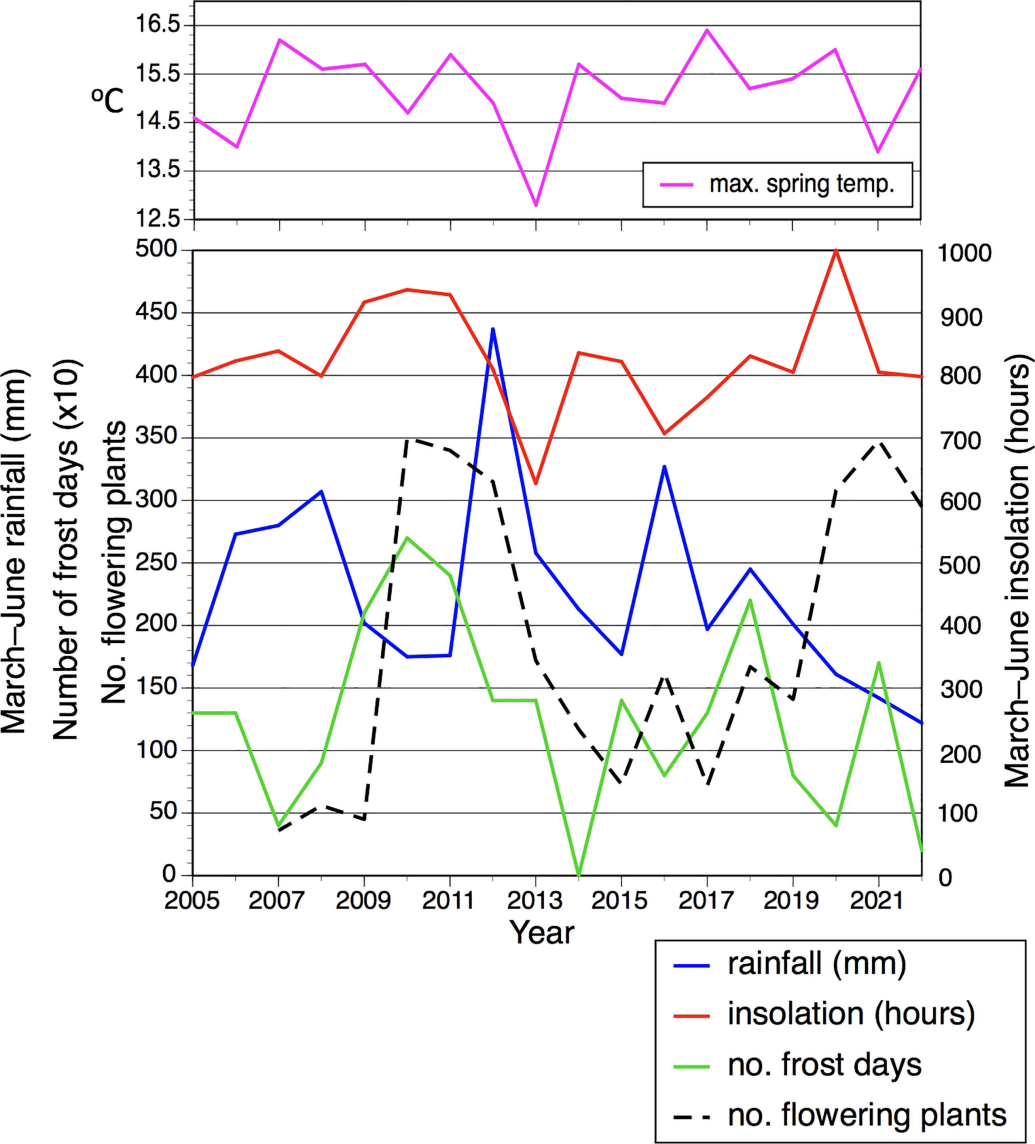
Spring rainfall, spring insolation, winter frost days, average spring maximum daily temperature (separate), and annual number of flowering plants in the Wolstonbury population of *Platanthera chlorantha*, each plotted as a time series between 2005 and 2022.

However, simply plotting annual number of flowering plants against March–June rainfall and insolation (**Figure 5**) for year t revealed more complex patterns not readily discerned from simple regression. Specifically, during the ten years of low to medium flowering the numbers of flowering plants tracked remarkably closely the spring rainfall figures (especially the period 2012–2020), whereas there is no discernible relationship during the two periods of three years when the number of flowering plants was unusually high. In contrast, numbers of flowering plants are not tracked by spring insolation. Nonetheless, it is striking that the first of the two three-year peaks in flowering is staggered one year after three consecutive spring sunshine figures that exceeded 450 hours and three consecutive winters in which the number of frost days exceeded 20 (**Table 1**; **Figure 5**). The only other spring exceeding 450 hours coincided with the first year of the second three-year flowering peak.

We speculated that prolonged winter frosts spanning the boundary between years t–1 and t might cause abortion of embryonic inflorescences developed during summer t–1 in order to reach maturity during June of year t, thereby reducing the number of plants successfully flowering that year. However, *r* values actually proved especially low for annual comparisons of frost days with all other variables in year t. Nonetheless, the number of frost days almost achieved statistical significance (*r* = 0.476, *p <*0.10) when compared at t–1 with the number of flowering plants (**Tables 1**, **4**); four of the six floriferous years (but only one less floriferous year, 2019) occurred *ca* 18 months after winters that featured 15 or more frost days (**Figure 5**). We also tested a prior hypothesis that comparatively large mean leaf area achieved by the June of year t–1 would preferentially resource the newly formed tuber, leading to more vigorous growth in year t that would be reflected in increased mean values for stem height and flower number. In fact, no such influence was detected, the highest such *r* value obtained being just 0.279 (results not tabulated).

**Figure 6:**
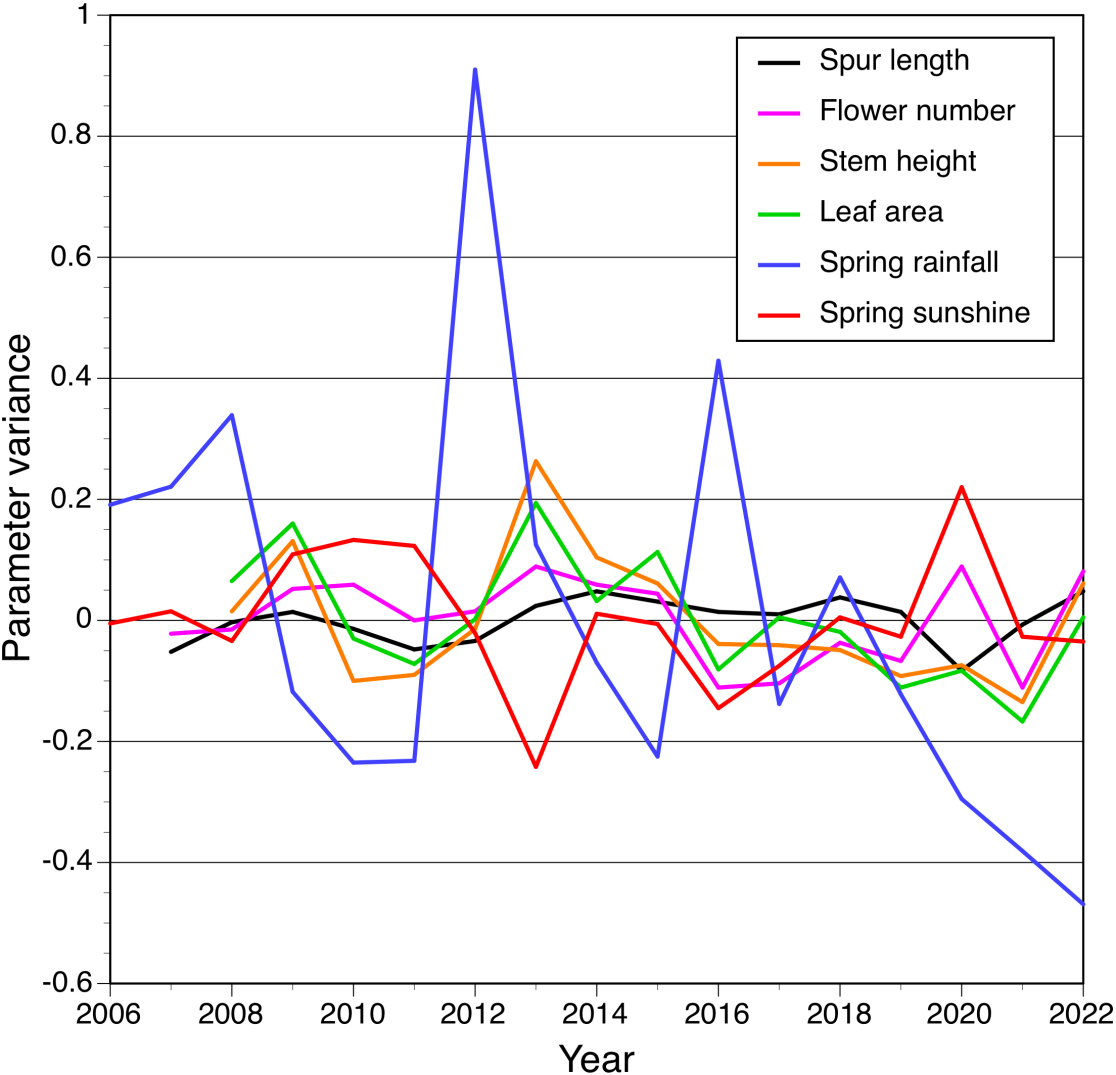
Cross-correlation of variance for the four morphological traits measured by us plus spring rainfall and insolation, each plotted as a time series between 2006 and 2022.

Similar comparisons made using cross-correlation techniques applied to variance (**Figure 6**) yielded encouragingly similar results, in that the same small subset of relationships between variables proved statistically significant: the positive correlations between rainfall and both stem height and leaf area for t–1 comparisons, and a less strong positive correlation between leaf area and stem height for year t (**Table 5**).

## Discussion

### The Wolstonbury population is representative of the species

Given that our study is confined to a single population of *P. chlorantha*, it is important that we demonstrate that it is typical of the species as a whole if our conclusions are to have more general validity. Certainly, the habitat – calcareous scrub and grassland – falls within the normal range. Admittedly, the absence of data for Wolstonbury plants on DNA-based properties or common-garden experiments means that we are unable to directly address issues such as plasticity, thus confining our comparisons to morphological traits.

Fortunately, we have access to directly comparable morphometric data gathered by Bateman et al. (2012) from 79 plants collectively representing 13 English populations of *P. chlorantha*. Comparing mean values for those other populations with the overall mean values for Wolstonbury summarised in **Table 1** shows acceptably close similarity: respective values are 32.5 mm (reduced to *ca* 30 mm if only open-habitat populations are considered) versus 29.0 mm for spur length, 12.5 versus 13.5 flowers per inflorescence, 36.7 mm versus 36.8 mm for stem height, and estimated leaf areas of approximately 60 cm^2^ versus 52 cm^2^. A further slight difference apparent in the data was that 3.8% of the plants measured by Bateman et al. (2012) bore three leaves rather than two, whereas such plants represented only 0.1% of the Wolstonbury population; there, 1.6% of plants bore only one leaf (**Table 1**).

It is also important to rule out the possibility that the Wolstonbury population could have been rendered atypical of the species through past hybridization. Although *Platanthera* L.C. Rich. is a genus of approximately 60–80 species (cf. Hapeman and Inoue, 1997; Bateman et al., 2009; Efimov, 2016), only two of those species are confirmed as occurring naturally in the British Isles (e.g. Bateman, 2022). Admittedly, they frequently hybridize when co-occurring (e.g. Mötlep et al., 2021), but the closest population of the second British species, *P. bifolia*, throughout the last quarter-century has lain 25 km to the west of Wolstonbury Hill (BSBI DDb database, consulted February 2023) – a distance sufficient to give confidence that the Wolstonbury population is genetically cohesive.

### Nature of the annual growth cycle

The morphology of an organism is dictated by its genetics as interpreted and modified through epigenetic and environmental factors (ecophenotypy) and through the degree of maturity and vigour already achieved by the organism (ontogeny).

**Figure 7:**
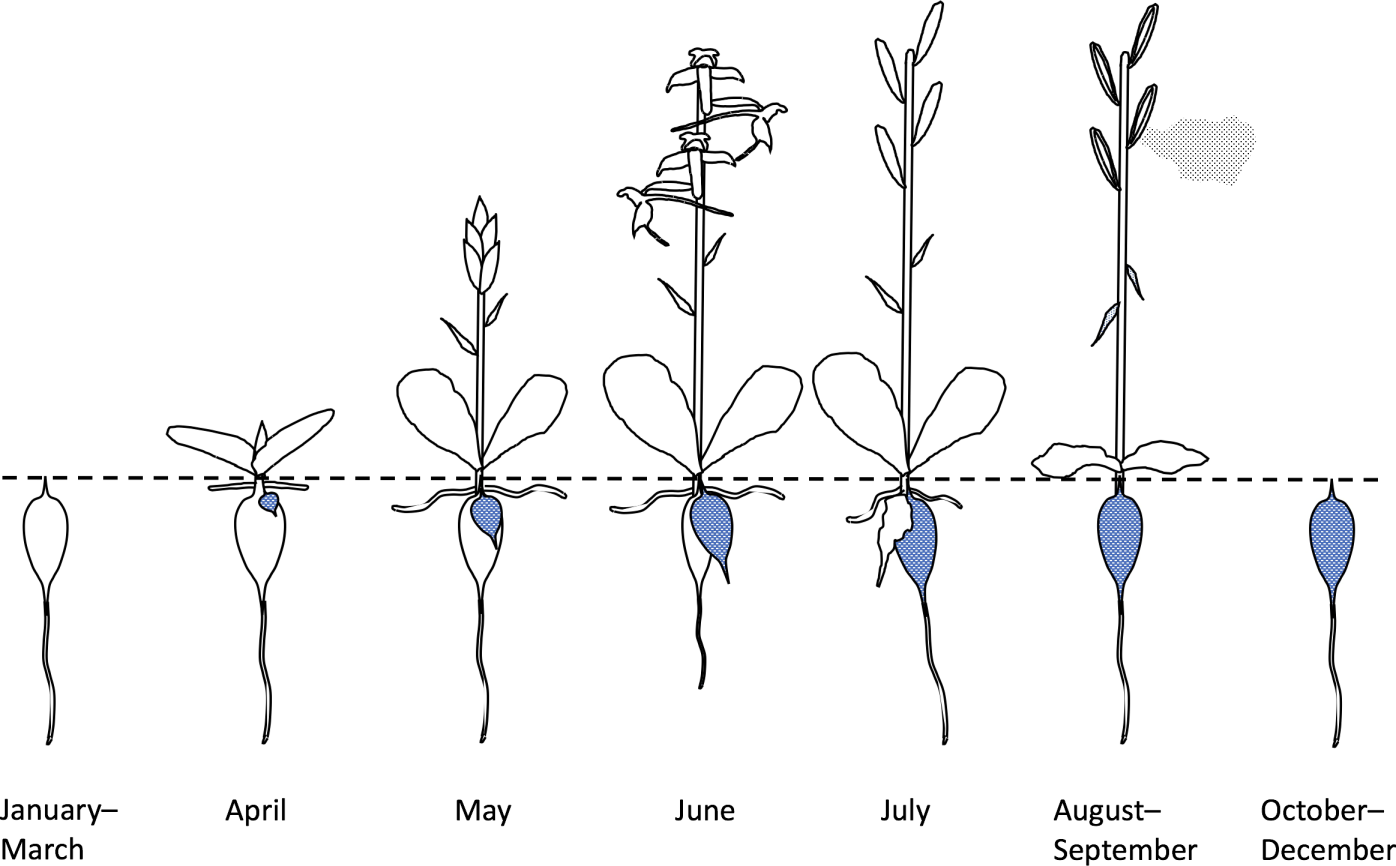
Annual life-history typical of *Platanthera chlorantha* in southern England. Note that March–June/July is the crucial period of investment in both the current year’s reproductive output and survival to the following year, achieved through somatic renewal as one or more replacement tubers (stippled).

In the present study, early stages in the life history from mycotrophic seed germination to mature tuber (e.g. Rasmussen, 1995) must be set aside, as our data relate only to mature individuals able to develop an inflorescence. Once having reached that stage of maturity, the Butterfly-orchid’s life history (**Figure 7**) is one of annual somatic replacement (e.g. Tatarenko and Kondo, 2003). Having over-wintered, the fusiform root-stem tuber produces a shoot that breaks ground in early spring, simultaneously generating a pair of basal leaves, and a radiating horizontal set of stem-roots, throughout the spring. At the same time, the over-wintered tuber begins to generate one or more new tubers. In England, plants that have “decided” to flower typically begin to elongate their stem above ground in April or early May; the shoot initially bears a very few small bract-like leaves before reaching anthesis in June. Successfully fertilized ovaries swell during June and July, at which point resourcing of the new root-stem tuber and its associated terminal root is also completed below ground. The ovaries dehisce their dust-seed in August and September, as that year’s vegetative growth – both above and below ground – withers and then gradually decays (**Figure 7**).

Obviously, the timing of annual measurement is important in ensuring consistency when gathering data on properties that are likely to differ only subtly from year to year. By the time we conducted our annual measurements in early–mid June (e.g. **Figure 1A**), leaf growth had ceased, and the number of flowers per inflorescence had long since been determined (see below). However, gradual stem growth continues during anthesis, as does spur length at a lower, more subtle level of variance (Bateman and Sexton, 2008; Bateman et al., 2012). These features therefore constitute a (slowly) moving target and require carefully timed measurement. A recent multifaceted study by Cullen et al. (2023) shows that early stages of spur elongations reflect enhanced rates of cell division but that the later stages that are of interest in the present study largely reflect cell elongation, supporting a conclusion made earlier by Bateman et al. (2012) using *Platanthera* as their preferred model.

### Floral variation is comparatively constrained

Coefficients of variation for the four traits measured here echo both relative and absolute values for these characters in previous morphometric studies of tuberous temperate orchids (e.g. Bateman and Rudall, 2011; Bateman et al., 2013).

The substantially lower coefficient of variation for spur length relative to the remaining three morphological traits reflects contributions from several different factors. Dimensions of the components of a flower are relatively highly constrained, as the flower is a disposable unit that reflects determinate growth and terminates a developmental cascade within the inflorescence. In addition, the relatively short period of the year allocated to flower development in general, and spur expansion in particular, limits opportunities for environmental modification of its mature morphology. Moreover, conventional wisdom states that the size of the overall floral display and the size and shape of those floral components that influence pollination success, notably the gynostemium and nectar-rich labellar spur, will be at least periodically subject to stabilizing selection, driven by the necessity of assuring frequent reproduction through seed dispersal (cf. Boberg and Ågren, 2009; Boberg et al., 2014; Ambroise et al., 2020; Brzosko et al., 2022). This innate conservatism inevitably limits variance in floral morphology, and therefore in our study it weakened all correlations that involved spur length – a phenomenon that is readily apparent in **Tables 2** **–** **5**.

Nonetheless, despite its comparatively low variance, spur length retains some residual interest. Previous studies of spur length in both *P. chlorantha* and *P. bifolia* (sister species to *P. chlorantha*: e.g. Bateman et al., 2009) showed that populations occupying shaded habitats have longer spurs than those in the open, and also revealed a latitudinal gradient of 2.2% increase in spur length per 100 km, as estimated along an approximate North–South transect from Scandinavia to North Africa (Bateman and Sexton, 2008; Bateman and Sexton, 2009; Bateman et al., 2012). Using data for 61 populations of *P. chlorantha* accumulated by Bateman et al. (2012), we can now demonstrate that labellar spurs of 21 populations growing in shaded habitats were on average approximately 12% longer than spurs of 40 populations occupying more open habitats – a figure that is reduced to 10% when adjusted for latitude. Indeed, this figure approximates the coefficient of variation calculated annually for the Wolstonbury population (**Table 1**), suggesting that preferred habitat has as great an influence on spur length as do the aggregate effects of innate properties such as tuber size and genetic composition.

We are unable to explain three statistically significant (albeit modest in scale) correlations involving spur length: the same-year positive correlation between spur length and stem height observed during six of the 15 relevant study years (**Table 2**; **Figure 2B**), and the apparent modest increases in stem height and flower number two years after a short-spur year (**Tables 2**, **3**; **Figures 3G, H** **)**. The equally modest but statistically significant negative correlation observed between spur length and spring insolation (**Tables 4**, **5**; **Figure 4A**) may relate to details of the process of spur growth, which is believed to reflect not only cell division but also cell elongation (Bateman and Sexton, 2008). The longer spurs of plants growing in shade suggest a minor, small-scale effect analogous with the etiolation observed in entire plants growing in shade. If so, this effect could represent the antithesis of the slightly reduced spur lengths evident in data for those years when the plants were subjected to unusually sunny springs.

### Plant vigour during a growing season appears largely predetermined

Within particular years, we expected a particularly strong positive correlation between stem height and flower number, as previous studies have suggested that in terrestrial orchids the proportion of the stem allocated to flower production is usually fairly consistent, measured at between one quarter and one third (e.g. Willems, 1978; Bateman et al., 2012; Bateman et al., 2021). Bateman et al. (2012) showed that the inflorescence of *P. chlorantha* averages a relatively stable 24 ± 6% of total stem length. Past studies of several other European orchid genera also show that spacing between flowers within inflorescences is fairly consistent among plants, thereby achieving an equally consistent floral display (e.g. Bateman et al., 2013; Bateman et al., 2021). We were therefore surprised to find that the inflorescence length–flower number relationship proved to be weaker on average than relationships between stem length and flower number or especially between stem length and leaf area (**Tables 2**, **3**; **Figures 2**, **3**).

Given that the leaves emerge before the stem expands to form an inflorescence, we also predicted a comparatively strong positive same-year relationship between leaf area and stem height, assuming that larger leaves would help to resource a taller stem and perhaps a larger number of leaves. This positive correlation was statistically strongly significant, both for individuals within years (**Figure 2F**) and population means analysed among years (**Figure 3A**). Wells and Cox (1989) were able to predict the likelihood of successful flowering of a plant of *Ophrys apifera* based on the number of rosette leaves that it produced (unlike *P. chlorantha*, its leaves – four or more in number in flowering individuals – are produced in the previous autumn). However, given that 98% of mature *P. chlorantha* plants bear two leaves, we were not afforded an opportunity to test this approach to predicting subsequent performance. Janeckova and Kindlmann (2002) found leaf area to be a strong predictor of whether individual plants of the terrestrial orchid Dactylorhiza fuchsii would flower during the following summer.

Overall, the behaviour of leaf area appears more complex than we initially anticipated. It was no surprise to find a strong positive same-year correlation between leaf area and stem height (**Figure 3A**), but the negative same-year correlation between leaf area and frequency of flowering (**Figure 3E**) demands a more complex explanation. We suspect that in years of few flowering plants, only the most vigorous plants that have produced the largest leaves are able to successfully generate flower spikes, whereas in fecund years, plants that are somewhat less well-resourced are also by some means encouraged to flower. In order to explore these intriguing issues further it would be necessary to measure leaf area of all the non-flowering plants present in the orchid population, in order to determine whether non-flowering plants that are relatively large-leaved in year t increase their tuber size and then proceed to flower preferentially in year t+1.

### Climate impacts population dynamics to dictate subsequent reproductive output

The low level of variation evident throughout the study period in mean spur length and mean flower number per inflorescence (**Table 1**) shows that there is little year-on-year difference in the amount of resources expended on reproduction by a typical flowering individual. It might be argued that contrasts among years in the availability of potential pollinators could nonetheless impact strongly on reproductive output. However, although this might be true of pollinator-limited orchids that rely on food deception or sexual deception, *P. chlorantha* offers a substantial nectar reward to any insect competent to access the apical half of the long labellar spur. Claessens and Kleynen (2011) listed as observed pollinators of *P. chlorantha* an impressive 29 species of 15 insect genera. They also reported fruit-set figures for 26 populations that we calculate collectively average 78 ± 13%. This figure is typical of food-rewarding (and also of autogamous) temperate-terrestrial orchid species, and is much higher than fruit-set frequencies calculated for orchid species that indulge in food deception or sexual deception (Neiland and Wilcock, 1998; Bateman, 2022). Substantial annual fluctuations in frequency of fruit-set are therefore unlikely.

Hence, we suspect that contrasts in annual overall reproductive effort are dictated almost entirely by the proportion of mature plants in the population that “choose” to flower in any particular year – a figure that proved highly variable at Wolstonbury (**Table 1**). This conclusion renders particularly intriguing our evidence that both relatively low spring rainfall and high spring sunshine strongly encourage plants to flower, but only when these environmental cues have been experienced by the plants during the previous spring (**Figures 4B, D** **)** when they were utilising the resources of the previous tuber (**Figure 7**). Destructive studies of numerous plants would be required in order to determine whether these apparently favourable climatic conditions in year t–1 lead to preferential resourcing of that year’s crop of replacement tubers, thereby increasing the total population biomass for deployment in year t.

The t–1 results highlighted in **Figure 4** do strongly indicate that the “decision” to flower is taken by a plant during formation of the new tuber, almost a year prior to its actual flowering. In this context, the embryonic inflorescence in *P. bifolia*, sister-species to *P. chlorantha*, was described by Tatarenko and Kondo (2003) as forming its inflorescence within the tuber during the autumn of the year that immediately preceded flowering. We have therefore initiated a programme of micro-dissection of early-stage above-ground shoots in the genus *Dactylorhiza*, which is closely related to, and has a very similar life history to, that of *Platanthera* (e.g. Bateman et al., 2018) but constitutes a more amenable experimental organism. It is possible that the number of flowers to be borne by the embryonic inflorescence is also determined during the previous summer.

If the commitment to flower is indeed made in the previous year, we suspected that a secondary “failsafe” mechanism might exist that offers the plant the ability to rescind that decision in the face of disadvantageous conditions encountered during the following spring. Such a mechanism would allow the plant to generate and retain leaves to aid immediately subsequent tuber formation but would permit early-stage abortion of the embryonic flowering stem under unsatisfactory environmental conditions. However, were this hypothesis correct, we would have expected to find at least occasional statistically significant same-year correlations between climatic variables versus stem height, flower number and/or leaf area, but we did not (**Table 4**).

An alternative mechanism of de facto abortion was reported by Sanger and Waite (1998) after monitoring a population of the tuberous European orchid *Ophrys sphegodes*. They observed frequent abortion of the entire above-ground parts of plants of prior to flowering in years when the population experienced suboptimal growing conditions during that same spring. Stroh (2019) suspected that similar casualties, perhaps caused primarily by frosts, occurred in his long-term study population of the orchid *Anacamptis morio*. Our approach to data collection was not designed to track premature leaf loss, which may similarly affect *Platanthera*, though the fact that *Ophrys* species produce leaf rosettes that differ from the paired leaves of *Platanthera* in being winter-green may increase their vulnerability to harsh winter climates.

### Might demographic recruitments trump environmental perturbations?

Such thoughts encouraged us to take one final look at our demographic data. Specifically, we find particularly remarkable the relationship through time between spring rainfall and flowering success that is evident in **Figure 5**. These graphs give the impression of a background state of comparatively low numbers of flowering plants broadly tracking spring rainfall. But two sudden radical rises in numbers of flowering plants are also evident, occurring ten years apart. Both are initiated in a relatively dry spring but then persist at a high level for two further years irrespective of amount of spring rainfall (compare the low rainfall of 2011 and exceptionally high rainfall of 2012, both accompanied by over 300 flowering plants). Do 2010 and 2020 – the years of exceptionally high recruitment to the cadre of plants successfully flowering – also represent years when there was a large-scale increase in the number of plants that possessed tubers sufficiently large to permit flowering? Is it even possible that the periodicity between the two phases of mass flowerings actually reflects successful establishment from seeds of large numbers of juvenile plants about ten years previously, stimulated by environmental conditions (more likely in the autumn than the spring) that proved unusually conducive to successful pairing of seeds with appropriate mycorrhizae and/or root-bacteria?

Unfortunately, our approach to data collection precluded estimation of the half-life of *Platanthera chlorantha*. Until a long-term demographic study is conducted that monitors specific plants from year to year, we cannot estimate the half-life of a *P. chlorantha* plant, which would have helped us to interpret the kind of complex patterns summarized in **Figures 5**, **6**. Data on broadly comparable species yielded surprisingly short half-life estimates of approximately two years for *Dactylorhiza viridis* (Willems and Melser, 1998), *Neotinea ustulata* (Tali, 2002) and *Ophrys sphegodes* (Hutchings, 2010). However, the majority of tuberous terrestrial orchid species – including *Orchis anthropophora*, *Spiranthes spiralis* (Wells, 1981) and *Ophrys apifera* (Wells and Cox, 1989) – approximate six to seven years. Our anecdotal observations on the Wolstonbury population would support a similar figure for *P. chlorantha*.

Our demographic investigation of *Platanthera chlorantha* makes an interesting comparison with that performed during the period 1966–1995 by Wells et al. (1998) on two populations of *Herminium monorchis*. Our results suggest that flowering of *Platanthera* is encouraged by low spring rainfall, high spring sunshine and perhaps also frequent frosts experienced in year t–1, whereas Wells et al. (1998) found that *Herminium* responded best to high rainfall, not only in year t–1 but especially in the subsequent year t (also responding positively to lower summer temperatures). *Herminium* favours habitats even more prone to desiccation than those occupied by *Platanthera*, and its flowering typically peaks one month later. Consequently, its two or three leaves often wither prior to flowering. And as already noted, tuberous orchid genera such as *Ophrys* and *Spiranthes* produce prostrate rosettes of several leaves during the autumn of year t–1 that are then obliged to withstand the succeeding winter conditions. When combined with different habitats preferences, such demographic contrasts are likely to strongly influence how particular species react to changing climate, suggesting that even vaguely accurate predictions of future behaviours will need to be species-specific.

### Long-term trends versus shorter-term crises

We concluded data collection for this study intrigued to discover whether a 16-year run of trait data would reveal any directional trends through time that would leave the population in a condition materially different from that in which it was first examined. But the only vaguely directional trend that we were able to identify appears trivial – specifically, the absence of years incurring unusually high coefficients of variation in flower number and leaf area during the last six years of the study. We suspect that a far longer period than 16 years is needed to modify a population through directional selection, and that the spotlight of selection rarely shines sufficiently long on any of the traits we have studied to influence their time-averaged mean values. The primary threats to the survival of plant populations such as Wolstonbury are more likely to be changes in their immediate environment. Some such changes would appear catastrophic, such as an intensive wildfire or a radical change in the dominant grazing regime. More gradual environmental changes such as increasing atmospheric CO_2_ levels would need to persist on a timescale of at least centuries in order to induce meaningful directional evolution.

In this context, we note with interest the strong, albeit largely indirect, influence of precipitation and insolation on reproductive success in our study population, and the similar inferences drawn from previous studies of genetic and epigenetic variation in other European orchids. As noted for an orchid genus closely related to *Platanthera* by Paun et al. (2010), “water availability in combination with temperature appears to be a key factor causing environmental allopatry in *Dactylorhiza*, being identified both at the epigenetic level and by transcriptome profiling” (as shown subsequently by Paun et al., 2011). Similarly, a recent study of the European rhizomatous orchid genus *Epipactis* by Evans et al. (2023) concluded that although a combination of temperature and altitude have strongly influenced genotypes close to the Mediterranean, further north it is precipitation that has been the primary influence. In summary, the current emphasis on research related to climate change appears justified.

## Conclusions

(1) The four morphological traits studied by us in the Greater Butterfly-orchid population at Wolstonbury were selected partly because we believed that they operated under contrasting levels of developmental constraint and vulnerability to ecophenotypic modification. This proved to be the case, as shown by consistent contrasts among traits in coefficients of variation, though annual fluctuations in the total number of plants flowering in the population showed much greater variance.
(2) The comparatively strong constraints on variation in spur length – a trait directly affecting reproductive competence – largely precluded statistically significant within-year correlations with the remaining traits.
(3) Comparison through the 16-year study period of mean annual values for traits with local meteorological data for that same growth period yielded only one statistically significant correlation – a modest reduction in the length of spurs produced at the end of relatively sunny springs. This observation accords with previous observations that plants that grow in shaded habitats bear somewhat longer spurs, and may reflect differential levels of hydration experienced during spur elongation once the bud has opened.
(4) In the case of all five of the remaining significant correlations detected, spring meteorological conditions in one year influenced the overall performance of the population during the following year. Comparatively high spring rainfall enhanced both leaf area and stem height a year later, whereas comparatively high spring insolation reduced stem height in the following year. Also, a remarkable dynamic was observed regarding number of flowering plants, wherein low rainfall and/or high insolation (possibly also frequent frosts) in year t–1 increased the proportion of plants flowering in the population in year t. These observations were confirmed through cross-correlation of standardised variance data.
(5) We are intrigued to note that the two phases of three successive years of intensive flowering observed in our study populations, beginning in 2010 and 2020 respectively, coincided with Southern Hemisphere transitions from El Niño to unusually deep La Niña conditions, which persisted for two and at least three years respectively (cf. Brönnimann et al., 2007; Zhang et al., 2014; National Weather Service Climate Prediction Centre, 2023). However, we accept that in this case, based on just 16 years of observation, correlation cannot necessarily be equated with causation.
(6) The various responses to t–1 climate are attributed to the complete somatic replacement inherent in the life-histories of the root-stem tubers – an ontogenetic property that characterises the majority of terrestrial orchids. The vigour and perhaps general performance of a plant during year t is dictated by the size and quality of the tuber formed 8–11 months earlier during year t–1.
(7) More intriguingly, each tuber apparently “decides” during year t–1 whether or not it will produce an inflorescence in year t, making that decision under the direct influence of year t–1 climatic conditions. It is possible that this decision can subsequently be modified through facultative abortion; this could either affect the embryonic inflorescence within the tuber during the winter or, more likely, the entire above-ground organs of the plant as they develop through the spring.
(8) Whether a decision that is taken by a plant almost a year before its crucial consequences can confer on that species any long-term adaptive advantage must surely rank as one of most fascinating (if least tractable) questions in evolutionary biology.
(9) No major directional trends in morphological traits or frequency of flowering were detected through the study period – the population reached Year 16 essentially unchanged from the condition that it exhibited in Year 1. Considering the present data from an evolutionary viewpoint, and placing them in the broader context of our previous studies on these and many other species of European orchids, we suspect that directional selection occurs only under circumstances when a population is influenced strongly, either directly or indirectly, by changes in its environment that either persist through long periods of time or are catastrophic.
(10) Current, albeit worryingly limited, evidence on the interactions of orchid species with climatic variables suggests that each species is likely to react differently to climate change. Important influencing factors include habitat preference, symbioses with pollinators and mycorrhizae, life-history properties such as the relative timings of leaf emergence and flowering, and demographic factors such as recruitment to the respective cadres of (a) juvenile survivors and (b) mature tubers sufficient in size to generate an inflorescence.
(11) We began synthesising our 16 years of data with several hypotheses that we wished to test, harbouring expectations of what the outcomes might prove to be. Those initial expectations have by no means always been met, while new questions have been raised. Further understanding of such medium-term population dynamics requires fusion of the multiple trait measurement approach taken here with the year-on-year monitoring of individual plants that has been adopted by several previous demographic studies. Although they remain a popular research topic, trends in reproductive output alone (in the case of *Platanthera chlorantha*, dominated by the decision whether to flower) are far from sufficient to explain overall population dynamics.

## Data availability statement

The raw data supporting the conclusions of this article will be made available by the authors, without undue reservation.

## Author contributions

Designed the project and gathered the data: DP and KS. Analysed the data: RB and DP. Wrote the manuscript: RB and DP. Edited and revised the manuscript: all authors. All authors contributed to the article and approved the submitted version.
